# Evaluation of Immune Response against Leishmaniasis in BALB/c Mice Immunized with Cationic DOTAP/DOPE/CHOL Liposomes Containing Soluble *Leishmania major* Antigens

**Published:** 2019

**Authors:** Mansure HOJATIZADE, Ali BADIEE, Ali KHAMESIPOUR, Mahmoud Reza JAAFARI

**Affiliations:** 1. Department of Basic Medical Sciences, Neyshabur University of Medical Sciences, Neyshabur, Iran; 2. Nanotechnology Research Center, Pharmaceutical Technology Institute, Mashhad University of Medical Sciences, Mashhad, Iran; 3. Center for Research and Training in Skin Diseases and Leprosy, Tehran University of Medical Sciences, Tehran, Iran; 4. Biotechnology Research Center, Pharmaceutical Technology Institute, Mashhad University of Medical Sciences, Mashhad, Iran

**Keywords:** *L. major*, BALB/c mice, *Leishmania* vaccine, Cationic liposomes, Dioleoyl-3trimethylammonium-propane

## Abstract

**Background:**

Whole killed *Leishmania* vaccine reached phase III clinical trials but failed to display significant efficacy in human mainly due to limited Th1 inducer adjuvant. Liposomes consisting of 1, 2-dioleoyl-3trimethylammonium-propane (DOTAP) bearing an inherent adjuvanticity and 1, 2-dioleoyl-L-α-glycero-3-phosphatidylethanolamine (DOPE) is well known to intensify the efficacy of positively charged liposomes.

**Methods:**

Soluble *Leishmania major* antigens (SLA) encapsulated in cationic liposomes using lipid film method in 2016). BALB/c mice were immunized subcutaneously (SC), three times in a 2-wk interval, with Lip (DOTAP)-SLA+, Lip (DOTAP/DOPE)-SLA+, Lip (DOTAP/DOPE/CHO)-SLA+, Lip (DOTAP/DOPE/CHO), Lip (DOPE/CHO), SLA or HEPES buffer. At week 2 after the last booster injection, immunized mice have challenged SC in the footpad with *L. major* parasites. To investigate the rate of protection and the type of immune response generated in mice, lesions development was assessed, IL-4 and IFN-γ levels with the ratio of IgG2a/IgG1 isotype were studied to describe the type of generated immune response.

**Results:**

Mice immunized with all liposomal form of SLA showed smaller footpad swelling and lower parasite burden in the spleen and footpad compared to the group of mice received buffer. However, these formulations did not show protection against leishmaniosis because of a generated mixed Th1/Th2 response in mice characterized by high production of IFN-γ and IL4 and a high titer of IgG1 and IgG2a antibody.

**Conclusion:**

Immunization with Lip (DOTAP/DOPE/CHO)-SLA+ was not an appropriate strategy to protect mice against leishmaniosis.

## Introduction

Leishmaniosis causes a spectrum of diseases ranging from self-healing cutaneous leishmaniosis (CL) to potentially fatal visceral form of leishmaniosis (VL) (1). CL is representing 50%–75% of all new instances. The number of CL cases is about 1 to 1.5 million annually, 90% of which is prevalent in several countries including Iran, Afghanistan, Saudi Arabia, Brazil, Peru and Syria (2).

*Leishmania major* is endemic in many rural areas of Iran, involving 17 out of 31 provinces (Khorasan Razavi, Khorasan Shomali, Khorasan Jonoobi ,Isfahan, Bushehr, Khuzestan, Ilam, Fars, Qom, Golestan, Yazd, Hormozgan, Kerman, Semnan, Sistan and Baluchistan, Lorestan and Tehran provinces), the foci of anthroponotic CL are active in some large cities and suburban areas of the country, such as Mashhad, Sabzevar and Neishabour (northeast); Kerman, Shiraz and Bam (south); and Kashan, Isfahan and Yazd (center) (3).

Recovery from CL results in the generation of long-lasting protection to further infection (4). Development of a practical vaccine is most achievable tool to control distinct forms of leishmaniosis. However, despite many attempts to developing a vaccine, currently, there is no vaccine against leishmaniasis (5). *Leishmania* antigens comprised of crude in the presence of an effective adjuvant induce a Th1 response and a degree of immunity in animal. Whole killed *Leishmania* vaccine reached phase III clinical trials but however failed to display significant efficacies mainly due to limited Th1 inducer adjuvant for use in human (6). Selected cationic lipids with an ammonium head group have immunostimulatory effect due to the innate positive charge and capacity to form bilayer structures which proffer efficient protein encapsulation and could be used as delivery systems in developing vaccine (7). DOTAP (1, 2-dioleoyl-3trimethylammonium-propane) liposomes act as a tool to raise the incompetently immunogenic profiles of peptide or protein antigens and induce CTL (Cytotoxic T lymphocyte) and Th1 responses (8). Moreover, cationic lipid DOPE is well known to intensify the transfection efficacy of cationic lipid-based formulations (9). Specific cellular signaling pathways and several kinases from the MAPK pathways are activated by cationic lipids. Activation of these kinases explains chemokines production and induction of CD80/CD86 cell surface expression (10). Induction of extracellular-signal-regulated kinase (ERK) phosphorylation was showed for DOTAP and diC14-amidine liposomes (11).

In the present study, SLA as a first-generation vaccine, encapsulated in positively charged liposomes and was used to immunize BALB/c mice. Then the type of induced response and the expanse of immunity were studied and compared with the control groups which received SLA alone or either buffer.

## Materials and Methods

### Parasites, SLA

SLA was prepared from *L. major* strain (MRHO/IR/75/ER) used for the preparation of experimental old world *Leishmania* vaccine and leishmanization in 2016 (12). The preparation of SLA was according to Scott et al protocol with minor modifications (13).

### Liposome preparation and characterization

Cationic liposomes with or without SLA were prepared by the film method, then the lipid film was hydrated in sterile buffer containing SLA (1 mg/ml) at 37 °C. The multilamellar vesicles obtained were converted to unilamellar ones using bath type sonicator. The empty liposomes were also prepared using the same procedure except the SLA was omitted.

### Size distribution and zeta potential analysis of nanoparticles

Dynamic Light Scattering Instrument was used to estimate the particle size distribution and zeta potential of the liposomes. Particle size was reported as the mean± standard deviation (n=3) and Zeta potentials were reported as the means ± zeta deviation (n=3).

### SDS-PAGE analysis of SLA and liposomal SLA

SDS-PAGE analysis of SLA and liposomal SLA were carried out to characterize and confirm qualitatively the concentration of antigen encapsulated in formulations containing SLA after purification by dialyzed against buffer. After electrophoresis, the gels were stained with silver for protein detection (14).

### Inoculation of BALB/c mice

Female BALB/c mice 6–8 wk’ old were carried out according to Ethical Committee Acts (Education Office dated March 31, 2010; proposal code 88527), based on the Specific National Ethical Guidelines for Biomedical Research issued by the Research and Technology Deputy of Ministry of Health and Medical Education (MOHME) of Iran.

All 7 groups of mice, 10 mice per group, were subcutaneously (SC) inoculated, three times in a 2-week interval, in the right footpad with either of the following formulations: Lip DOTAP 4 μmol / DOPE 4 μmol / SLA 1mġ/ml, Lip DOTAP 4 μ mol / DOPE 4 μmol / CHOL 4 μ mol / SLA 1mġ / ml, Lip DOTAP 4 μ mol/ SLA 1mġ / ml, Lip DOTAP 4 μ mol / DOPE 4 μmol / CHOL 4 μ mol, Lip DOPE 4 μ mol / CHOL 4 μmol, SLA 1mġ / ml, HEPES-sucrose buffer (10 mM, 10% w/v, pH 7.5)

### Challenge with L. major

At week 2 after last booster injection, the immunized mice were challenged SC in the left footpad with 1×10^6^ *L. major* isolated at stationary phase in 50 μL volume. Lesion progress was recorded in each mouse by assessment of footpad swelling using a metric caliper. Grading of wound size was done by subtracting the diameter of the uninfected contra-lateral footpad from that of the infected one.

### Quantitative parasite burden after challenge

The number of live parasites in the infected footpad and spleen of mice was evaluated using limiting dilution assay method as described in the past (15). Briefly, the mice were sacrificed at week 8 post-challenge; the spleens and the infected footpad tissues in each group were homogenized in media and were cultured in plates (16). Presence and absence of motile parasites were checked by an inverted microscope. The number of motile promastigotes was defined by Graph Pad Prism software.

### Antibody isotype assay

Two weeks after the last booster injection before challenge and at week 8 after challenge blood samples were isolated from mice and the sera were collected to evaluate anti-SLA IgG1, IgG2a and IgG total antibodies by ELISA method (15).

### Enzyme-linked immune spot (ELISPOT) assay

At 10 wk after the last booster injection, 3 mice from each group were sacrificed and the spleens were aseptically removed and homogenized. Then, the isolated spleen cells were used according to the manufacturer’s instructions. Spot counting was done with a Kodak 1D software package.

### Statistical analysis

To assess the significance of the differences between various groups One-way ANOVA statistical test was used. In the case of significant F value, Tukey–Kramer multiple comparisons test was carried out as a post-test to compare the means in different groups of mice. *P*<0.05 was considered as statistically significant.

## Results

### Liposome characterization

**Fig. 1:**
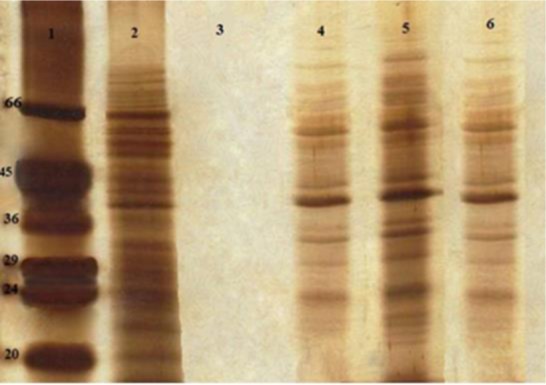
SDS-PAGE analysis of SLA and liposomal SLA. Lane 1, Low-range protein standard (Sigma, USA), Lane 2 SLA (10 μg), Lane 3 Lip (DOTAP/DOPE/CHOL). Lane 4 Lip DOTAP)-SLA (2.5 μg), Lane 5 Lip (DOTAP/DOPE)-SLA (5 μg), Lane 6 Lip (DOTAP/DOPE/CHOL)-SLA (2.5 μg)

According to Table 1, liposomes containing SLA showed a positive zeta potential and the mean diameters of Lip-SLA+ formulations were around 500 nm and the mean diameters of empty liposomes were around 150 nm. SDS-PAGE analysis of soluble SLA and liposomal SLA revealed several protein bands with a range of molecular weight between 10 to 70 kDa (Fig. 1).

**Table 1:** Particle size distribution, Polydispersity index (PDI) and zeta potential of various liposomal formulations

***formulations***	***Size (nm)***	***PDI***	***Zeta potential(mv)***
Lip (DOTAP/DOPE)-SLA+	458±38	0.44±0.02	30 ±3.6
Lip (DOTAP/DOPE/CHOL)-SLA+	493±25	0.35±0.01	35 ±2.1
Lip (DOTAP)-SLA+	578±24	0.36±0.03	29 ± 2.5
Lip (DOTAP/DOPE/CHOL)	130±6.11	0.23±0.02	72 ±2.6
Lip (DOPE/CHOL)	170±12.4	0.18±0.006	60 ± 2.3

(mean ± SD, n = 3)

### Challenge results

**Fig. 2:**
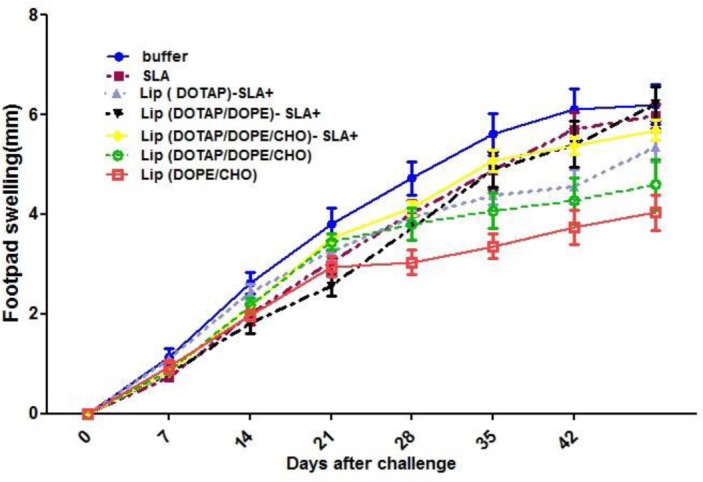
Footpad swelling in mice immunized subcutaneously .The footpad thickness was measured weekly for 6 wk. Each point represents the average increase in footpad thickness± SD (n=7)

Lesion development was recorded by weekly measurement of footpad thickness (Fig. 2). At week 3 after challenge The lesion size in mice immunized with Lip(DOTAP/DOPE)-SLA+ was still significantly (*P*<0.05) smaller than the buffer group, also the mice immunized with Lip(DOTAP)-SLA+ was significantly (*P*<0.05) smaller lesion size at week 5 after challenge compared to buffer group.

The lesion size progressed at a more rapid rate in control groups which received either buffer or SLA than in mice immunized with Lip (DOPE/CHOL) or Lip (DOTAP/DOPE/CHOL) (*P*<0.01) at week 6 after challenge. The lesion size in mice immunized with SLA progressed continuously and no protection was detected in this group. At week 8 after challenge, progress in lesion size was slower in mice immunized with Lip (DOPE/CHOL) or Lip (DOTAP/DOPE/CHOL) compared with the mice that received Lip SLA+, the footpad swelling in this group of mice was significantly (*P*<0.01) smaller than the group that received buffer or SLA. In different groups, the footpad swelling reached a plateau after 6 wk (Fig. 2), but the disease was progressed by metastasis to spleen and foot and the mice lost their foot.

### Parasite burden in foot and spleen

**Fig. 3:**
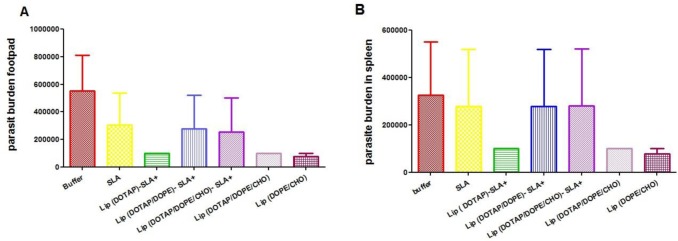
**A:** Footpad parasite burden in mice. A limiting dilution analysis was performed at week 8 after the challenge on the footpads of different groups of mice and cultured in tetraplicate in 8-fold serial dilutions. The number of viable parasite per foot was enumerated. The bar represents the average score ± SEM (n = 3) **B:** splenic parasite burden in mice. A limiting dilution analysis was performed at week 8 after the challenge on the spleen of different group of mice and cultured in tetraplicate in 8-fold serial dilutions. The number of viable *L. major* was determined in the spleen of different groups of mice and the number of viable parasite per foot was enumerated. The barre presents the average score ± SEM (n = 3)

At week 8 after challenges, the number of *L. major* in the footpads of mice immunized with Lip SLA+, SLA or buffer was more than the groups received Lip (DOTAP/DOPE/CHO), Lip (DOPE/CHO) (Fig. 3A). Mice immunized with Lip (DOTAP/DOPE/CHO), or Lip (DOPE/CHO) showed the lower number of live parasite in the spleen of compared with the other groups but there was no significant difference between those groups (Fig. 3B).

### Antibody response

**Fig. 4:**
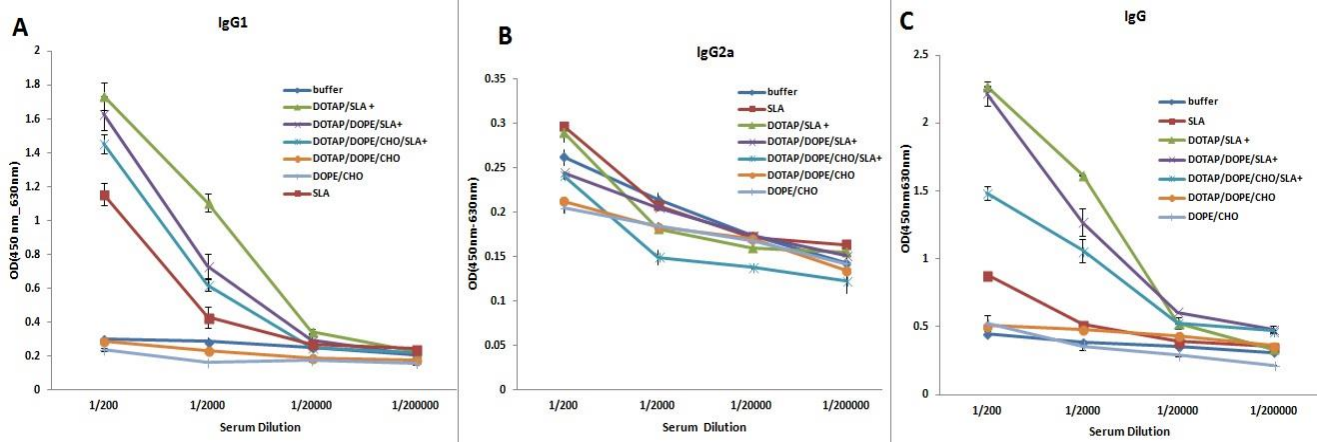
Levels of anti-SLA IgG1 (A), IgG2a (B), and IgG (C) in pooledsera of mice before challenge. Blood samples were collected from the mice 2 wk after the last booster injection and before challenge. The serum samples were pooled. The anti-SLA IgG1, IgG2a, and IgG titers were determined using ELISA method. The assay was performed in triplicate at 200, 2,000, 20,000, or 200,000-fold dilution for each sample. Values are the mean ± SD

**Fig. 5:**
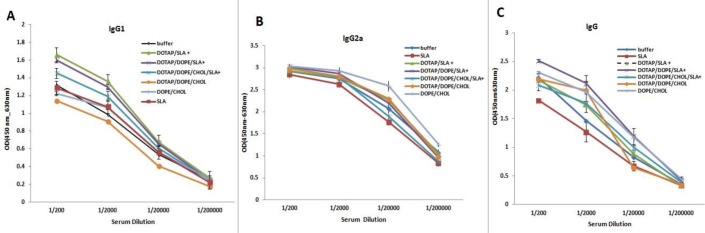
Levels of anti-SLA IgG1 (A), IgG2a (B), and IgG (C) in pooledsera of mice after challenge. Blood samples were collected from the mice 10 wk after the last booster injection and after challenge. The serum samples were pooled. The anti-SLA IgG1, IgG2a, and IgG titers were determined using ELISA method. The assay was performed in triplicate at200, 2,000, 20,000, or 200,000-fold dilution for each sample. Values are the mean ± SD

The anti-SLA IgG antibodies and IgG1, IgG2a subclasses were titrated before (Fig. 4A–C) and after (Fig. 5A–C) challenge. The sera of mice immunized with Lip-SLA+ before challenge showed significantly (*P*<0.001) higher levels of IgG total antibodies compared to control groups in serum dilution of 1:200 (Fig. 4C). In terms of IgG1, the level of IgG1 in sera of mice immunized with Lip SLA+ was significantly (*P*<0.05) higher than the group received buffer in serum dilution of 1:200 or 1:2000 (Fig. 4A).

Challenge with *L. major* induced elevation of IgG1, IgG2a, and IgG antibody levels in nearly all the groups of mice compared with before challenge (Fig. 5A–C). Moreover, the sera of mice immunized with Lip (DOPE/CHOL) or Lip (DOTAP/DOPE/CHOL) showed significantly (*P*<0.03) elevated levels of IgG2a antibodies compared with the control groups in serum dilution of 1:20000 (Fig. 5B). After challenge, the sera of mice immunized with Lip-SLA+ showed significantly a higher level of IgG1antibody compared with buffer group (*P*<0.05) in serum dilution of 1:200 (Fig. 5A).

Generally, all groups showed significantly (*P*<0.001) elevated levels of IgG total antibodies compared with the SLA groups in serum dilution of 1:200 (Fig. 5C).

### In vitro IFN-γ and IL-4 assay

**Fig. 6:**
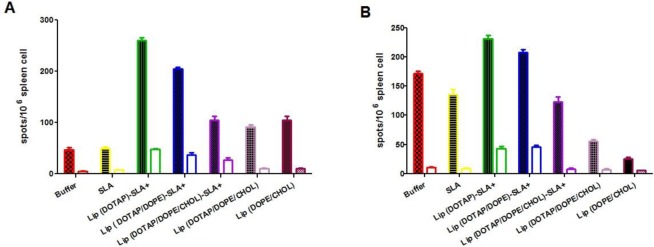
Cytokine levels in immunized mice at week 2 after the last booster injection. Mononuclear splenocytes were cultured in the paresence of SLA (10 μg/ml) and amounts of IFN-γ (A) or IL-4 (B) were detected using ElISPOT method. Results are shown as Mean ± SEM (n=3) and expressed as spot-forming units (SFU) per 10^6^splenocytes, white columns are negative control without any stimulation.

At week 10 after the last booster injection, splenocytes were isolated and restimulated in vitro by concanavalin A, SLA or media to check IFN-γ and IL-4 concentration. The results of ELISPOT assays showed that mice immunized with Lip-SLA+ showed a higher level of IFN-γ and IL-4 comparison to those immunized with other formulation of vaccine (Fig. 6). Further, there was no significant difference between the groups of mice received either SLA or Lip-SLA+ in terms of IFN-γ or IL-4 secretion.

## Discussion

In the present study, SLA was used as a first-generation vaccine. Effective vaccines against leishmaniosis require an extensive range of protective epitopes which cover a wide range of MHC types in a populace (17). Based on leishmanization results, crude *Leishmania* antigens are considered promising candidate for preparation of vaccine (18). However, first-generation vaccines in phase III trials revealed little efficacy partially due to the lack of a suitable adjuvant (19).

Previously, in order to enhance the immunogenicity of autoclaved *Leishmania major* vaccine (ALM) + BCG, ALM was adsorbed to aluminum hydroxide, a single dose of alum-precipitated autoclaved *Leishmania major* vaccine (ALM) mixed with BCG showed to be safe and decreased the incidence rate from 12 to 3.7% (20). In another study, to determine the acceptability and toxicity whole killed *L. major (KLm*), it’s given as an intradermal injection. Findings showed higher doses of the vaccine, up to l000μg produced no unacceptable side effects and addition of 400μg KLm vaccine to a normal dose of BCG produces reactions not acceptable and the major side effects and toxicity were related to the use of BCG (21).

Indeed, choice of a suitable adjuvant is crucial for nearly any modern vaccine to generate effective immune responses (22). Present study was designed to explore whether cationic DOTAP/DOPE/CHO liposomes act as an immunoadjuvant and appropriate delivery system for SLA to generate a strong Th1 response and protection against *L. major* challenge in murine model of leishmaniosis.

In current study DOTAP was used in liposome structures to use inherent adjuvanticity of the quaternary ammonium combination, also to employ positively charged liposomes which interface more efficiently with SLA and the anionic species on the APC s ′ cell surface and so to enhance antigen delivery to dendritic cells (DC), DOTAP act as an stimulator of DC that activated extracellular-signal-regulated kinase (ERK) and chemokine induction (11). DOTAP induced migration of activated DC to the draining lymph node (DLN) and efficiently provoke functional antigen-specific CTL responses. Reactive oxygen species generated by DOTAP cationic lipid in DLN showed a possible mechanism of the beginning interaction between DOTAP and DC (23). In addition, DOTAP liposomes also induced transcription of several inflammatory chemokines (11). We have earlier assessed the role of DOTAP in liposomal formulation to enhancement of immune response against leishmaniosis, our findings point out that DOTAP in association with 1mg/ml SLA elicit Th1 response and a degree of protection in murine model of leishmaniosis (24). DOTAP have 2 unsaturated bonds in its structure which make a low transition temperature (0 °C) and liposomes unstable in vivo, and cholesterol can be used as a membrane stabilizer in liposome formulation (25). Adding cholesterol maximize the in vivo stability of the liposomes that lead to a more rigid formation which enhances stimulation of CTL responses (26). Another purpose for using cholesterol was to boost cytoplasmic release of the antigens and prevent the vesicle lysosomal break down (27). Moreover, lipid: DOPE (1:1) combinations were fit to form stable liposomes (9).

A major factor that influences the behavior of liposomes after interstitial injection is particulates size (28). Large particles induce IL-12 production but small size particles do not induce IL-12 production (29). Liposomes 250–700 nm in diameter induced the greatest IFN-γ secretion by restimulated spleen cells and elicited a Th1 response and increases both accumulation in the draining lymph nodes and persistence at the injection site (30). We found previously that the small size vesicles (<200 nm) do not protect BALB/c mice against leishmaniosis, but the large size vesicles (400 nm or larger) protect animals (31). Table 1 shows that Lip-SLA+ formulations have an appropriate size of around 500 nm.

The results of ELISPOT assays showed that spleen cells isolated from the mice immunized with Lip-SLA+ released higher amounts of IFN-γ (Fig. 6A) in comparison with the groups of mice immunized with buffer or SLA. Moreover, high amounts of IL-4 (Fig. 6B) observed in these groups. Mice immunized with these formulations of Lip-SLA+ showed a mix Th1/Th2 response with no effective responses against leishmaniosis. Generation of Th1/Th2 responses by SLA immunization is consistent with the previous studies since BALB/c mice have a natural tendency to develop Th2 responses (32). The current data surprisingly showed that mice immunized with Lip (DOPE/CHOL) or Lip (DOTAP/DOPE/CHOL) in absence of SLA induced a Th1 responses and a degree of protection based on the results of lesion size and the number of live *L. major* in footpad and spleen. In addition, the ratios of IgG2a/IgG1 (a marker for the induction of Th1-like response) in sera of mice immunized with these formulations of vaccine were higher than the other groups (Table 2).

**Table 2:** The ratio of IgG2a: IgG1 before and after challenge at different serum dilutions

***Groups***	***Serum dilution***							
	***1/200***		***1/2000***		***1/20000***		***1/200000***	
	***Before***	***After***	***Before***	***After***	***Before***	***After***	***Before***	***After***
Buffer	0.86	2.20	0.74	2.79	0.68	3.88	0.68	4.24
SLA	0.25	2.21	0.48	2.45	0.63	3.14	0.67	3.77
Lip (DOTAP)-SLA+	0.16	1.76	0.16	2.04	0.46	3.32	0.68	4.73
Lip (DOTAP/DOPE)-SLA+	0.15	1.88	0.28	2.21	0.58	3.32	0.73	3.89
Lip (DOTAP/DOPE/CHOL)-SLA+	0.16	2.06	0.24	2.33	0.54	3.13	0.55	3.57
Lip (DOTAP/DOPE/CHOL)	0.74	2.61	0.79	3.07	0.89	5.67	0.76	5.63
Lip (DOPE/CHOL)	0.85	2.47	1.12	2.75	0.79	4.57	0.91	4.87

(mean ± SD, n = 3)

However, recent study indicated that cationic lipids in absence of antigens do promote specific immune response (11). Further research should be done to investigate whether positively charged liposomes act as an appropriate delivery system and effective immune adjuvant to induce a Th1 response and protection against infection diseases where we need a Th1 responses, particularly against leishmaniosis.

## Conclusion

Immunization with Lip (DOTAP/DOPE/CHO)-SLA+ was not an appropriate strategy to protect mice against leishmaniosis.
